# PathVisio 3: An Extendable Pathway Analysis Toolbox

**DOI:** 10.1371/journal.pcbi.1004085

**Published:** 2015-02-23

**Authors:** Martina Kutmon, Martijn P. van Iersel, Anwesha Bohler, Thomas Kelder, Nuno Nunes, Alexander R. Pico, Chris T. Evelo

**Affiliations:** 1 Department of Bioinformatics - BiGCaT, Maastricht University, Maastricht, The Netherlands; 2 Maastricht Centre for Systems Biology (MaCSBio), Maastricht University, Maastricht, The Netherlands; 3 General Bioinformatics, Reading, United Kingdom; 4 EdgeLeap B.V., Utrecht, The Netherlands; 5 Gladstone Institutes, San Francisco, California, United States of America; Carnegie Mellon University, United States of America

## Abstract

PathVisio is a commonly used pathway editor, visualization and analysis software. Biological pathways have been used by biologists for many years to describe the detailed steps in biological processes. Those powerful, visual representations help researchers to better understand, share and discuss knowledge. Since the first publication of PathVisio in 2008, the original paper was cited more than 170 times and PathVisio was used in many different biological studies. As an online editor PathVisio is also integrated in the community curated pathway database WikiPathways.

Here we present the third version of PathVisio with the newest additions and improvements of the application. The core features of PathVisio are pathway drawing, advanced data visualization and pathway statistics. Additionally, PathVisio 3 introduces a new powerful extension systems that allows other developers to contribute additional functionality in form of plugins without changing the core application.

PathVisio can be downloaded from http://www.pathvisio.org and in 2014 PathVisio 3 has been downloaded over 5,500 times. There are already more than 15 plugins available in the central plugin repository. PathVisio is a freely available, open-source tool published under the Apache 2.0 license (http://www.apache.org/licenses/LICENSE-2.0). It is implemented in Java and thus runs on all major operating systems. The code repository is available at http://svn.bigcat.unimaas.nl/pathvisio. The support mailing list for users is available on https://groups.google.com/forum/#!forum/wikipathways-discuss and for developers on https://groups.google.com/forum/#!forum/wikipathways-devel.

This is a *PLOS Computational Biology* software article.

## Introduction

*A picture says more than a thousand words*. For many years biologists have been drawing pathway diagrams to gain a better understanding of the underlying biology. These diagrams are found everywhere: in textbooks, research articles, posters, lab journals or presentations and they have proven themselves as powerful tools to organize, share and discuss knowledge. Pathway diagrams have also become immensely useful for computational analysis and interpretation of large-scale experimental data when properly modelled. Complex diseases like cancer or heart failure are known to be caused by malfunctioning pathways instead of individual genes, so the study and collection of biological pathways is crucial to get insights into complicated disease mechanisms. Nowadays, computers allow researchers to use tools to draw pathway diagrams that are much more than just pictures; they contain annotations, literature references and comments for each element and interaction in a pathway. These enriched pathway diagrams open the possibilities to perform advanced pathway analysis and data visualization to get a more comprehensive understanding of experimental data.

In 2008, we presented the first version of our pathway visualization and analysis tool PathVisio [1]. Since then, PathVisio has been used in numerous studies to draw biological pathways, perform pathway statistics or visualize biological data on pathways [2–10].

PathVisio has undergone active development and grown beyond a simple tool into a comprehensive and extendable pathway analysis toolbox. Besides its standalone graphical desktop version, PathVisio is often used as a library to read, write, store, convert and model pathway information. It is also used in different websites and workflows to act as a pathway editor and visualization tool. For example, a light-weight applet version of PathVisio is integrated in the community curated pathway database WikiPathways [11] and ProfileDb, a resource for proteomics and cross-omics biomarker discovery, uses PathVisio to visualize differential expression results on pathway diagrams [12].

In previous versions PathVisio provided a simple but limited interface for extensions through plugins. A plugin is a small software component that adds a specific feature to an existing application. In the case of PathVisio, a plugin could provide for example a new statistical method, a new drawing standard or additional information about elements in the pathway. The usage of available plugins enables users to refine the pathway analysis workflow in PathVisio and build an application with all the necessary modules relevant for their research.

Here we introduce the third version of the pathway visualization and analysis tool PathVisio. The aim is to present the newest additions and improvements of the application, especially the new plugin extension system, as well as the plugin repository and the integrated plugin manager. PathVisio is a freely available, open-source tool allowing independent developers to contribute plugins to provide new functionality. PathVisio is implemented in Java and thus runs on all major operating systems. The focus of this new version of PathVisio lies on modularity, extensibility and improved usability.

## Design and Implementation

In the last six years, PathVisio has been substantially extended and the core application was refactored using the OSGi framework (Open Service Gateway initiative) to achieve a better, modular system that can be easily extended with so called plugins [13]. OSGi also allows plugins to depend on each other to avoid code redundancy and promote code reusability. Such modular systems keep the core of an application stable and maintainable while the functionality can be easily extended allowing users to build an application designed for their needs [14].

First, we will discuss the new modular structure of PathVisio 3, then the plugin repository will be introduced, and last the usability and advantages of the new plugin manager will be shown.

### Modularisation with OSGi

**Fig 1:**
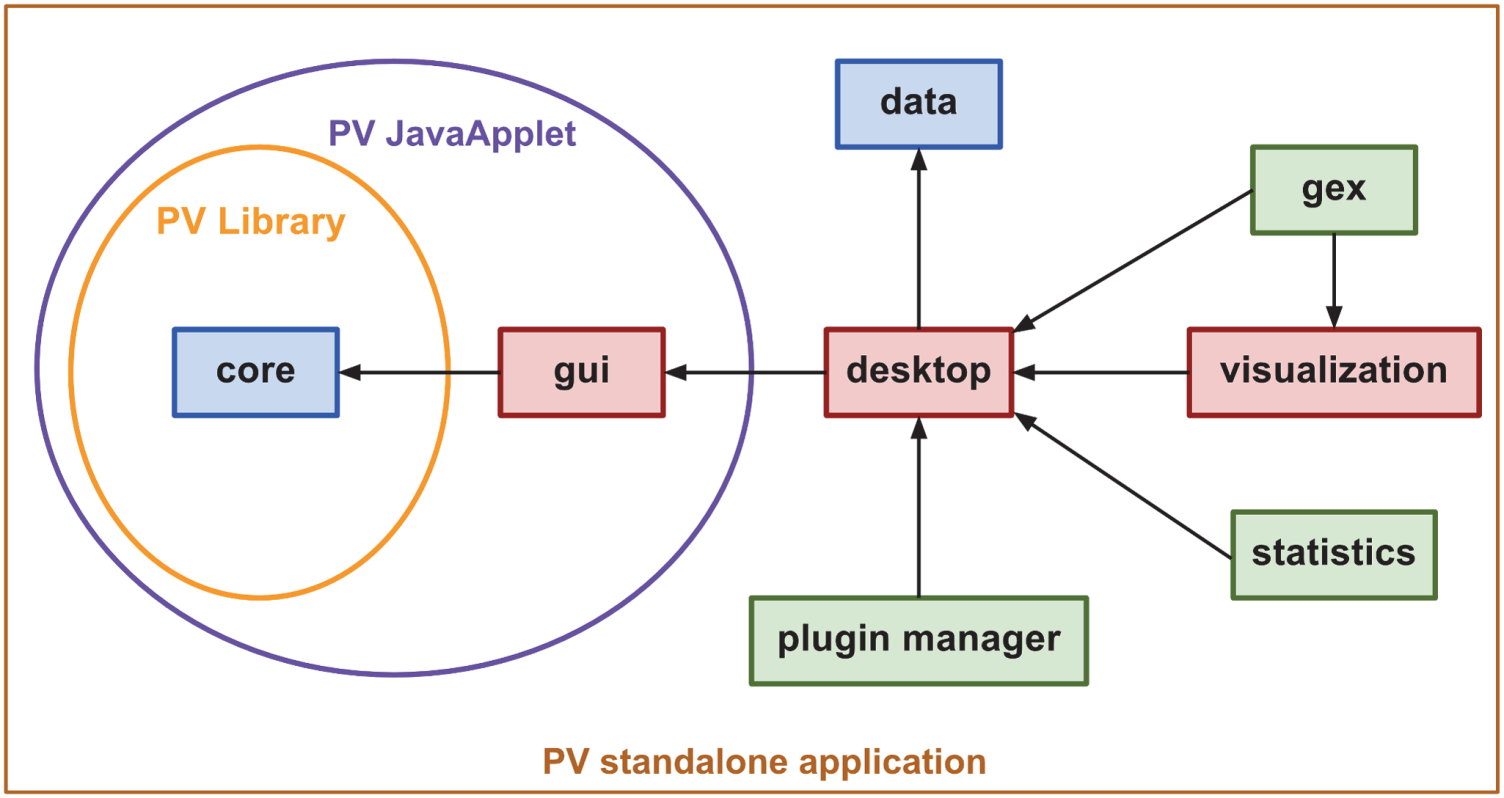
Transitive dependency structure of PathVisio 3. The application consists of eight modules each providing specific functionality. The modules *core* and *data* are independent modules (colored in blue) that function as libraries that can be reused outside of PathVisio (PV). Especially the *core* module is often used as a PV library for reading and writing of pathway files. Other modules in red, *gui*, *desktop* and *visualization*, provide functionality that is used by other modules. Green modules, *gex*, *statistics* and *plugin manager*, are not used by other PV modules but can be used by PV plugins. The PV JavaApplet version integrated in WikiPathways uses the *core* and *gui* modules.

PathVisio 3 consists of eight OSGi modules that build the core application, each being responsible for one crucial part of the application. As illustrated in Fig. 1, the modules nicely separate the different parts of the application.

The *core* module of PathVisio 3 contains the non-user interface backend, including the data model, import and export functionality and general settings and preferences. This module can also be used as a library by other software tools for reading, editing and writing pathway files in PathVisio’s native GPML (Graphical Pathway Markup Language, http://www.pathvisio.org/gpml) format. The *gui* (graphical user interface) module implements the basic user interface which is shared between the standalone and the applet version of PathVisio. The applet version is integrated in WikiPathways as an online pathway editor. The more advanced, full-powered graphical user interface for the standalone application is provided by the *desktop* module. It is also the central connecting point for plugins. The *plugin manager* module handles the connection to the plugin repository as well as installing and uninstalling plugins. The *gex* module contributes the functionality for importing experimental data together with the *data* module which defines the interfaces for storing and handling experimental data. The *visualization* module then provides a simple but flexible way to visualize the experimental data on the data nodes in the pathways. To identify significantly altered pathways in an experimental dataset, the *statistics* module contributes a standard over-representation analysis algorithm based on a hypergeometric test [15].

### PathVisio plugin repository

The new PathVisio plugin repository consists of two separate parts, (i) the repository itself which stores all necessary plugin files as well as their dependencies and (ii) the PathVisio plugin database and front-end.

The PathVisio repository is located at http://repository.pathvisio.org. It contains all plugin files and third-party dependencies. The RepoIndex library (https://github.com/osgi/bindex) builds a complete dependency structure of the repository and writes it in an XML file named repository.xml.

The PathVisio plugin database is an independent mySQL database containing location information and metadata, e.g. description, authors and release notes, about each plugin. The database is integrated into the WordPress framework (http://wordpress.org/) to take advantage of some of the built in functionalities of WordPress, like capabilities to tag, browse, search, comment and evaluate plugins.

### Plugin manager

To make it easier for users to find and install plugins, PathVisio 3 incorporates a plugin manager that connects to the repository and enables a one-click installation of plugins from within the application. The plugin manager allows users to browse plugins by categories and provides additional information about the plugin when selected, like description or author information.

**Fig 2:**
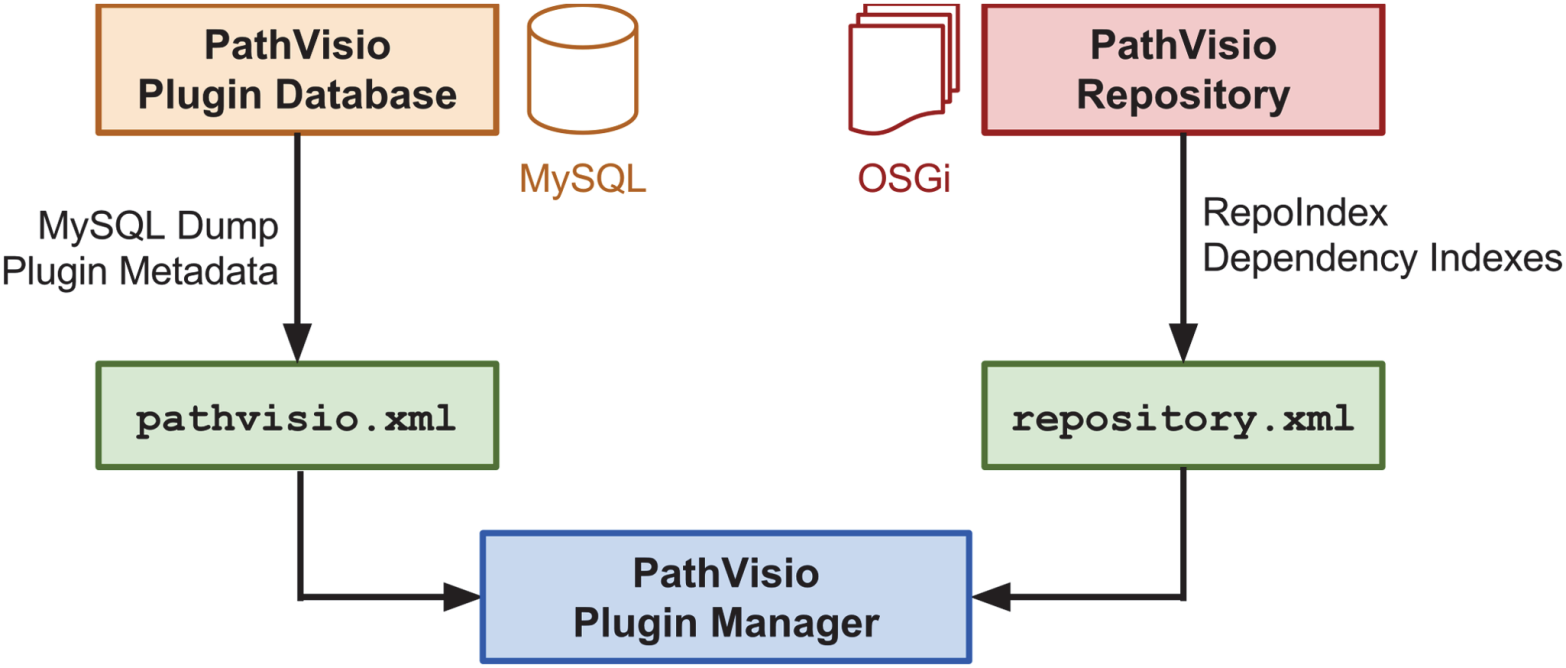
Plugin extension and installation system of PathVisio 3. The plugin repository stores all plugin files and their dependencies. The RepoIndex library is used to create a repository.xml file which contains the dependency indexes of all plugins. Metadata about plugins is stored in the PathVisio plugin database which is then exported into a pathvisio.xml file. The PathVisio 3 plugin manager retrieves data from both files to facilitate the installation of plugins in PathVisio 3.

Fig. 2 shows the connections between the different components that are used by the plugin manager. This new plugin manager module retrieves data from two different files, the repository.xml file and the pathvisio.xml file. The repository.xml file is created by the RepoIndex library and stores the complete dependency structure of the repository. Additional metadata about the plugin, like developers, description or categories, are retrieved from the pathvisio.xml file which is created from the PathVisio plugin database.

Consequently, the new extension system takes care of the installation of plugins and all required dependencies. If a plugin depends on another plugin or a third party library, the plugin manager makes sure that all required OSGi bundles are downloaded, installed and started. Therefore the complex dependency structure is hidden from the user and installation is much easier and faster.

## Results

PathVisio has been used in a substantial number of publications in the last six years and the analysis workflow has been further developed and improved. PathVisio 3 also provides several interfaces allowing plugins to integrate tightly into the application. The new plugin repository and manager finally bring the functionality of the plugins to all users by offering a simple and user-friendly interface for plugin installation.

In this section, we will first highlight the new features of PathVisio in an updated feature table, then the standard pathway analysis workflow in PathVisio will be demonstrated and we will show how plugins can hook into the application and provide new functionality to the user.

### Feature table

In Table 1 the most important features of PathVisio 3 are summarized. A feature comparison table with other tools like Vanted [16], ProMeTra [17], KEGG Atlas [18] or Ingenuity [19] is available in S1 Table.

**Table 1:** PathVisio 3 feature table.

**Feature**	**Description**
File import	Default: GPML (http://www.pathvisio.org/gpml/) Plugins: MIMML ([24], MIM plugin), SBGNML ([23], SBGN plugin), SBML ([31], PathSBML), BioPAX ([22], BioPAX plugin), gene list (MAPPBuilder)
File export	Default: GPML, PNG, PDF, SVG, TIFF, Eu.Gene [32], datanode list Plugins: MIMML (MIM plugin), SBGNML (SBGN plugin), SBML (PathSBML), HTML (HTMLexporter), BioPAX (BioPAX plugin)
Pathway drawing standards	Default: Basic GPML style Plugins: SBGN, MIM
Identifier mapping	Integrated BridgeDb framework [20] for advanced identifier mapping for pathway elements and interactions in the pathways. All major database identifiers including probe ids for genes, proteins and metabolites are supported.
Pathway statistics	Default: Over-representation analysis (Z-Score) Plugins: Gene set enrichment analysis (GSEA plugin)
Data visualization	Pathway nodes: gradient-based visualization for numeric data, rule-based visualization for numeric and nonnumeric data Interactions: color and line thickness visualization (IntViz plugin)
Plugin extension system	Plugin manager allows one-click installation of plugins from central plugin repository to enable additional features.
Pathway database connection	WikiPathways: searching, browsing, updating, uploading biological pathways (WikiPathways plugin)
Workflow integration	The core module can be used as a library to read, write, store, convert and model pathway information. Calling PathVisio functionality from other programming languages through XML-RPC server (PathVisioRPC)
Online data access	Several plugins provide connections to other online resources to give more information about the individual elements in the pathway, like BiomartConnect about gene products, MetInfo about metabolites or PathwayLoom about known interaction partners.

### Pathway analysis workflow in PathVisio

The core application has three main features: (1) pathway drawing, (2) data visualization and (3) pathway statistics. The integrated identifier mapping framework BridgeDb [20] allows pathway authors to annotate the elements in their pathways with their identifier system of choice and automatically takes care of the mapping when e.g. experimental data with another identifier system is loaded.

The data visualization and pathway statistics modules have been first introduced in PathVisio 2 and further improved and extended in PathVisio 3.

**Fig 3:**
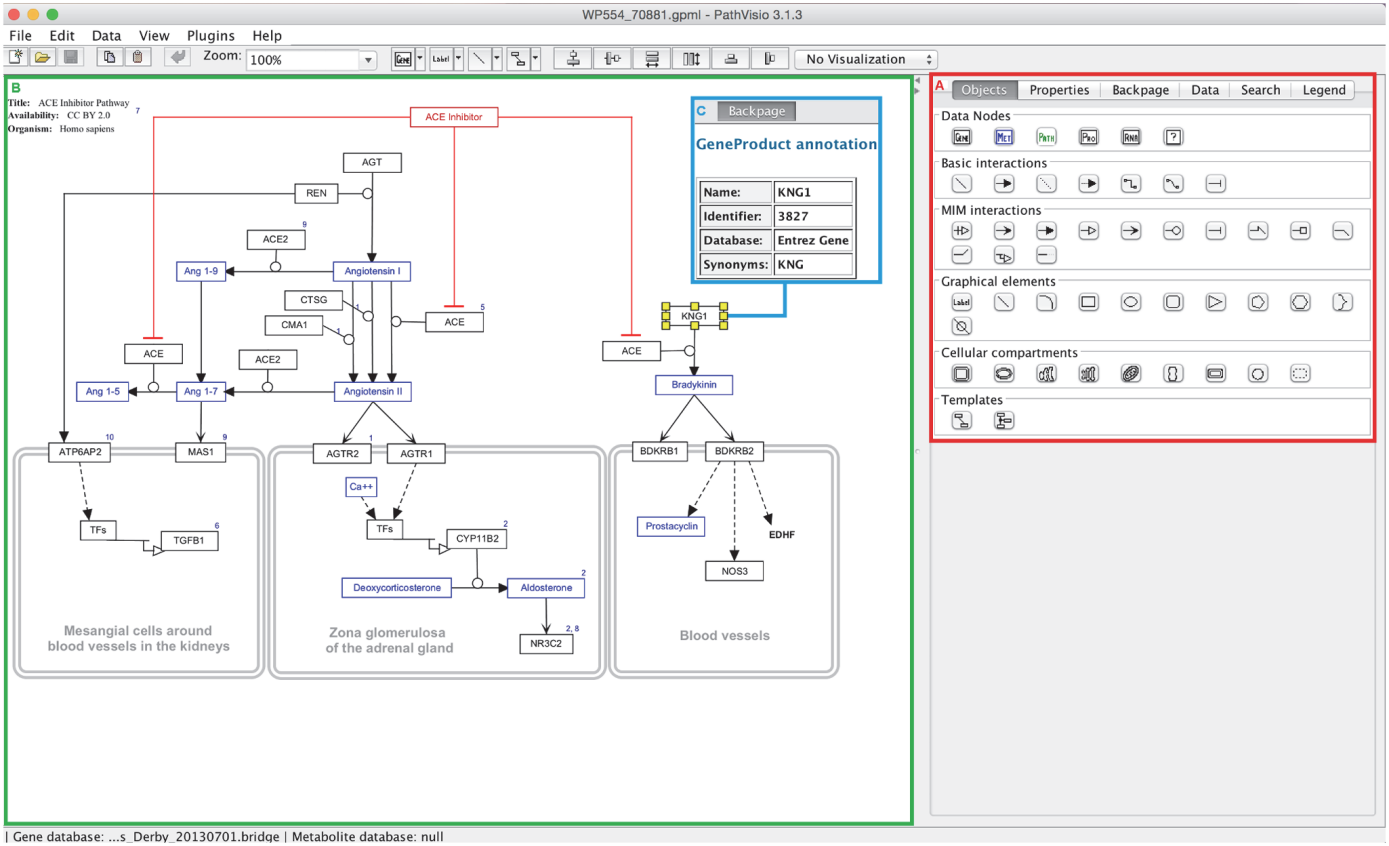
PathVisio 3, a full-powered pathway editor. (A) The basic drawing palette contains data nodes, interactions, graphical elements, cellular compartments and a few templates. Simple drag-and-drop mechanism allows users to add the elements in the pathway diagram. (B) The ACE inhibitor pathway on WikiPathways (http://www.wikipathways.org/instance/WP554) was drawn in PathVisio describing the downstream effects of angiotensin-converting-enzyme (ACE) inhibtors. (C) The entities and interactions in the pathways can be annotated with external identifiers. In this example the pathway author annotated the *KNG1* gene with the Entrez Gene identifier 3827. PathVisio utilizes the BridgeDb identifier mapping framework to free the user from manual identifier mapping steps.

**Pathway drawing**. Biological pathway diagrams represent the sequence of events in biological processes. They often contain different biological entities, like genes, proteins or metabolites, and interactions between them, like conversion, stimulation or inhibition. As illustrated in Fig. 3, PathVisio is a full pathway editor which allows users to draw the biological events, add graphical elements like shapes or labels and annotate all the biological entities and interactions with external database identifiers. The drag-and-drop mechanism for adding new elements is used similar as in PowerPoint and other drawing tools. Besides the external database annotation, users can also add publication references to each entity or interaction in the pathway establishing the pathway as a complete literature reference collection for the biological process described.

**Data visualization**. The visualization of experimental and other data is a crucial aspect in the analysis and investigation of biological pathways. PathVisio allows users to import their experimental data and visualize it on the data nodes and interactions in the pathway. The integrated identifier mapping framework takes care of mapping the data points to the intended pathway elements, therefore the user is not restricted to a specific identifier system. In integrative studies, transcriptomics, proteomics and metabolomics data can be visualized simultaneously to provide a more complete view of the underlying biology [6].

**Fig 4:**
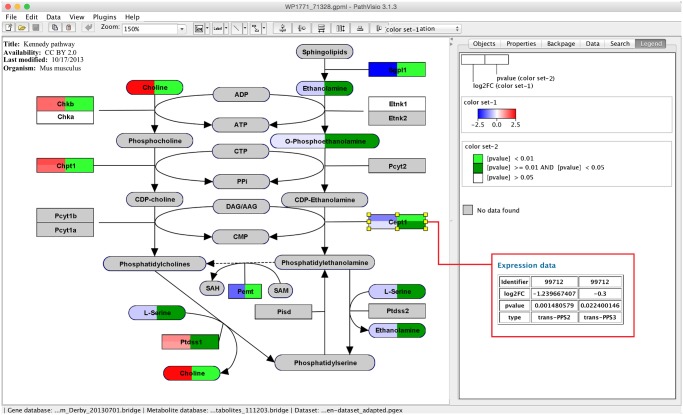
Multi-omics visualization in PathVisio. Two transcriptomics datasets are visualized together with a metabolomics dataset on the Kennedy pathway from WikiPathways (http://www.wikipathways.org/instance/WP1771). The log2FC is visualized in the first column of the data node boxes using a gradient from blue over white to red. In the second column three levels of p-values are visualized (p-value < 0.01, < 0.05 and > 0.05). The expression data for a selected gene or metabolite is shown in the “Data” tab on the right side. In the red rectangle the expression data for the selected *Cept1* gene is shown. There are two measurements for the gene from the two transcriptomics datasets, therefore the gene box in the pathway is split horizontally into two rows.

As detailed in Fig. 4, the visualization interface in PathVisio enables users to visualize multiple data points on the data nodes in the diagram. The boxes are split up in separate columns and for each column the user can define a gradient or color rule visualization. A gradient is used for a continuous visualization of numeric values like the log2FC or an activity measurement in an experiment. The color rules are used to define colors for discrete categories like p-value levels (p-value < 0.01, p-value < 0.05, p-value > 0.05). The example dataset visualized in Fig. 4 is a combined dataset of two transcriptomics and one metabolomics experiments. The first column in the datanode boxes represents the log2FC and the second column the p-value. The log2FC is visualized with a gradient from blue over white to red, while the p-value is visualized with a discrete color rule. If the dataset contains multiple measurements for one data node, the box is split horizontally into separate rows each representing one measurement.

The visualization options in PathVisio 3 can be used to visualize time-series data (one column for each time point) [2], tissue expression comparisons (one column for each tissue) [21] and other complex multi-omics experiments.

**Pathway statistics**. The goal of pathway statistics is to find pathways that are altered in an experimental dataset. The basic pathway statistics implementation in PathVisio is an over-representation analysis based on the statistical methods used in the MAPPFinder tool [15].

**Fig 5:**
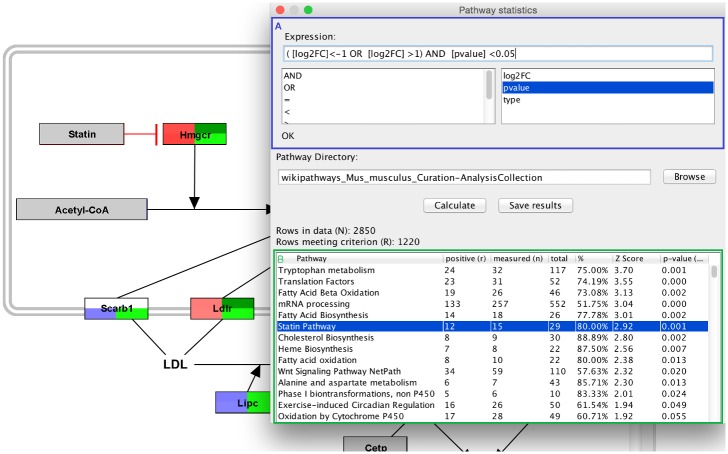
Pathway statistics result in PathVisio. The user defines the criterion for significantly changed genes with an absolute log2FC > 1 (A). A Z-Score is calculated for each pathway in the pathway collection and in the result table the pathways are ranked based on their Z-Score (B). A high Z-Score indicates that the pathway is more affected than expected based on the overall dataset. The user can click on each pathway to open the pathway with the data visualized on it.

First, the user defines a criterion to select the differentially expressed genes in the dataset. In Fig. 5A, the criteria filters genes with an absolute log2FC > 1 and a p-value < 0.05. The mouse pathway collection from WikiPathways was downloaded and selected.

The statistics module calculates the total number of genes measured in the dataset (N) and the number of genes meeting the criterion (R). All genes in N and R are present in at least one pathways. Genes that are not found in any pathway are ignored in the analysis. The Z-Score is calculated for each pathway in the collection. Therefore the statistics module counts the total number of elements in the pathway (total), the number of genes in the pathway measured in the experiment (measured → n) and the number of genes in the pathway meeting the criterion (positive → r) (see Fig. 5B).

A commonly used score for over-representation analysis is the Z-Score. The Z-Score is the score calculated by a standard statistical test under the hypergeometric distribution. It indicates if a particular pathway shows a difference in the ratio of genes meeting the criterion as compared to the complete dataset. It is calculated by subtracting the expected number of genes meeting the criterion from the observed number divided by the standard deviation of the observed number of genes:

$$\begin{array}{c} \text{Z-Score}=\frac{\left(r-n\frac{R}{N}\right)}{\sqrt{n\left(\frac{R}{N}\right)\left(1-\frac{R}{N}\right)\left(1-\frac{n-1}{N-1}\right)}} \end{array}$$

The pathways are ranked based on their Z-Score. A positive Z-Score indicates a pathway with more genes meeting the criterion than expected based on the complete dataset. A negative Z-Score indicates that less genes meet the criterion than expected. In the example in Fig. 5 pathways with a high Z-Score have more significantly up- or down-regulated genes than expected. Therefore those processes are highly affected in the experiment and should be further analysed. Over-representation analysis does not take the pathway topology into account, so it is important that the users look at the pathway diagrams by clicking on the rows in the table and visualize the experimental data on the diagram to interpret the biological outcome.

### Plugins in PathVisio

PathVisio 3 provides a powerful and flexible way for plugins to integrate new functionality into the application. The variety of plugins shows that PathVisio can be extended in a lot of different ways and although initially PathVisio started as a pathway editor, it grew into an advanced and extendable pathway visualization and analysis toolbox.

The implementation of different pathway related standards is crucial to fulfil the requirements of a state-of-the-art pathway editor. BioPAX is a standard language to exchange biological pathway data [22]. The BioPAX3 plugin allows users to import and export pathways in BioPAX level 3 which is the latest release of the BioPAX format. Furthermore, there are two plugins providing functionality to draw pathways in the commonly used SBGN (Systems Biology Graphical Notation [23]) and MIM (Molecular Interaction Maps [24]) drawing standards. The PathVisio-Validator plugin [25] assists users in creating biological pathway diagrams with the SBGN or PathVisio-MIM [26] plugins. It validates the diagrams and highlights possible warnings and errors in the pathway.

Pathway databases still only cover 48% of all human protein-coding genes (see S2 Table). Therefore the creation and curation of biological pathways is still of high importance. Recently we released the WikiPathways plugin for PathVisio which enables users to search and browse the database directly from within PathVisio but also allows the uploading and updating of pathways through the standalone pathway editor. Integrating this functionality in PathVisio 3 enables pathway curators to use all the available plugins while creating new pathways or curating existing ones. Since the release of this plugin several curation related plugins have been developed to facilitate the curation of the WikiPathways pathways. Furthermore plugins focussed on data integration can be used to facilitate the exploration and understanding of biological pathways. As an example, the pathway curator could use the PathVisio-Faceted Search plugin [27] to integrate experimental data and data from publicly available online resources. Another useful plugin is PathwayLoom which provides known interaction partners for a selected node in the pathway. This can help the curator to select the next element in the process.

Also the integration of additional data about the elements in the pathway is useful when creating and curating biological pathways. The BiomartConnect plugin queries the Ensembl database for additional information about gene products, like chromosomal position, %GC content or known variants. The MetInfo plugin provides more data about the metabolites in a pathway, like InChI key or predicted MS and NMR peaks. Plugins connecting to UniProt, Protein Data Bank (PDB) and interaction databases are under development.

### Integration of PathVisio in workflows and other applications

To enable the integration of PathVisio in an automated workflow, we developed PathVisioRPC (http://projects.bigcat.unimaas.nl/pathvisiorpc/) to be able to call PathVisio from other programming languages through an XML-RPC server. It enables users to programmatically draw pathways, visualize data on pathways and perform pathway statistics. This is especially convenient and time-saving when studying multiple datasets or datasets with many different comparisons.

Furthermore PathVisio is often used as a library to read, write, store, convert and model pathway information. The nice separation of the different modules in PathVisio 3 enables developers to integrate this functionality in other application simply by including the *core* module of PathVisio 3. This module is also used in the WikiPathways App for Cytoscape 3 [28]. Cytoscape is a popular network analysis and visualization tool [29] and the WikiPathways app allows users to load pathways as networks in Cytoscape to perform network analysis.

### Pathvisiojs: a JavaScript version of PathVisio

The pathvisiojs JavaScript library is a diagram viewer (implemented and available on WikiPathways) and editor (under development) for biological pathways. The viewer converts GPML source data into JSON for easier handling in JavaScript and then renders it as an SVG image in the users browser. The result is an interactive and searchable image with external reference linkouts via BridgeDb [20]. In future releases, more advanced editing functionalities are planned based on those available in the PathVisio desktop application.

## Availability and Future Directions

PathVisio 3 is a freely available, open source pathway editor, visualization and analysis toolbox implemented in Java. It runs on all major operating systems as a Java webstart program or as a binary installation.

- Download: http://www.pathvisio.org/downloads/
- Documentation and tutorials: http://www.pathvisio.org
- Instructions for core and plugin developers: http://developers.pathvisio.org
- Plugin repository: http://www.pathvisio.org/plugins/plugins-repo/
- Source code: http://svn.bigcat.unimaas.nl/pathvisio/, see S1 Code.
- Integrated identifier mapping framework: BridgeDb (http://www.bridgedb.org)
- Pathvisiojs code repository: https://github.com/wikipathways/pathvisiojs

### Future directions

Future development will focus on (1) more advanced pathway analysis methods, (2) improved data integration and visualization and (3) automated update mechanisms.

**(1) Advanced pathway analysis methods**. The default pathway analysis method in PathVisio 3 is a simple over-representation analysis. Users can also use the Gene Set Enrichment Analysis (GSEA) plugin which implements a functional class scoring method which does not require a specific threshold for splitting up significant and nonsignificant measurements. This method uses all the molecular measurements and their expression levels. The next step for PathVisio is the implementation of an topology-based pathway analysis method. While over-representation analysis and functional class scoring only consider the number of genes in the pathways, topology-based methods also look at the interactions between the elements in the pathways [30].

**(2) Improved data integration and visualization**. PathVisio 3 supports the visualization of transcriptomics, proteomics and metabolomics data on the elements in the pathways. Recently a plugin has been developed to allow visualization of fluxomics data on the interactions in the pathways. Integration of other experimental data like genetic variation, methylation or phosphorylation states is needed to be able to study biology in all its complexity. For most of these additional data types new advanced visualization methods are needed.

**(3) Automated update mechanisms**. In the next major release of PathVisio, we are planning an automated update mechanism for the main application and the installed plugins. The application can be upgraded as soon as a new release is available. We will provide installers for all major operating systems that will facilitate the installation of new PathVisio versions.

## Supporting Information

S1 Code Source code of PathVisio version 3.1.3. (RAR)

S1 Table Pathway tools comparison. (PDF)

S2 Table Gene coverage in pathway databases. All numbers were calculated with the BridgeDb mapping database build on 1 July 2013 (Hs_Derby_20130701.bridge → http://bridgedb.org/data/gene_database/). We used the Reactome, KEGG and WikiPathways webservices to retrieve the gene lists and map them all to Ensembl identifiers. We only included genes that can also be mapped to UniProt to focus on protein coding genes. This gives a basic indication of the gene coverage in the pathway databases. The scripts for the calculations can be downloaded from https://github.com/mkutmon/wp-scripts/blob/master/PathwayResourceGeneCoverage/src/org/wikipathways/Stats.java
(PDF)

S1 Text Installation instructions for PathVisio 3. (PDF)

S2 Text Tutorials and example data. (PDF)
