# Supplementary material for: PathVisio 3: An Extendable Pathway Analysis Toolbox
Source: PLoS Comput Biol. 2015 Feb 23;11(2):e1004085. doi: 10.1371/journal.pcbi.1004085 (PMC4338111; doi:10.1371/journal.pcbi.1004085)
Supplement: S1 Table — (PDF) [file pcbi.1004085.s002.pdf]

S1 Table
Manuscript: PathVisio 3: An extendable pathway analysis toolbox
Authors: M. Kutmon, MP van Iersel, A Bohler, T Kelder, N Nunes, AR Pico, CT Evelo

	Website	License	Type	Operating Systems	Pathway Import Formats	Pathway database, Knowledge Base
**PathVisio 3**	http://www.pathvisio.org/	free software (Apache 2.0)	desktop application or applet	Windows, Mac OSX, Linux	GPML, BioPAX, SBGN, SBML, MIM	Databases: WikiPathways, Reactome, NetPath, Wormbase, BioModels
**Vanted**	http://vanted.ipk-gatersleben.de/	free software (GPLv2)	desktop application	Windows, Mac OSX, Linux	GML, KGML, SBML2, PAJEK	KEGG, Biomodels
**ProMeTra**	https://prometra.cebitec.uni-bielefeld.de	no license specified	web application	Windows, Mac OSX, Linux	SVG	Only user uploaded pathway maps some converted from KEGG or other resources
**KEGG Atlas**	http://www.genome.jp/kegg/atlas.html	no license specified	web application	Windows, Mac OSX, Linux	only KEGG pathways can be used	KEGG Pathways
**Ingenuity**	http://www.ingenuity.com/	commercial software	desktop application or applet	tested for Windows and Mac	SBML, BioPAX, SIF, XGMML, PSI-MI	Ingenuity knowledge base

Table S1A. Pathway tool comparison table.

	Pathway creation	File export	Experimental Data	Data Type	Identifiers Supported
**PathVisio 3**	Intuitive, simple creation of fully annotated pathway diagrams Support of different drawing standards: SBGN, MIM	GPML. BioPAX, SBGN, SBML, MIM, PNG, SVG, TIFF, PDF, Gene list, Eu.Gene	Transcriptomics, proteomics, metabolomics, fluxomics	numerical and non-numerical data	advanced identifier mapping framework integrated (all major identifier systems are supported)
**Vanted**	Graph-Editor - flexible tool to change the shape, color, size and other attributes of nodes and edges	GML, JPEG, PNG, SVG, PDF	Transcriptomics, proteomics, metabolomics	numerical data	Identifiers used in the networks (KEGG Compound and EC numbers for KEGG networks)
**ProMeTra**	Pathway creation as SVG in InkScape	SVG	Transcriptomics, proteomics, metabolomics	numerical data	KEGG compound, locus tags, gene names
**KEGG Atlas**	-	PNG	Transcriptomics, proteomics, metabolomics	defining color directly	KEGG compound, EC number
**Ingenuity**	Creating new and extending existing pathways is possible	JPEG, GIF, PNG, HTML, SVG, EPS, EMF, BMP, PDF, TIFF	Transcriptomics, proteomics, metabolomics, microRNAs, SNPs	numeric data	most major identifier systems are supported

Table S1B. Pathway tool comparison table.
