# Supplementary material for: PathVisio 3: An Extendable Pathway Analysis Toolbox
Source: PLoS Comput Biol. 2015 Feb 23;11(2):e1004085. doi: 10.1371/journal.pcbi.1004085 (PMC4338111; doi:10.1371/journal.pcbi.1004085)
Supplement: S2 Table — All numbers were calculated with the BridgeDb mapping database build on 1 July 2013 (Hs_Derby_20130701.bridge → http://bridgedb.org/data/gene_database/). We used the Reactome, KEGG and WikiPathways webservices to retrieve the gene lists and map them all to Ensembl identifiers. We only included genes that can also be mapped to UniProt to focus on protein coding genes. This gives a basic indication of the gene coverage in the pathway databases. The scripts for the calculations can be downloaded from https://github.com/mkutmon/wp-scripts/blob/master/PathwayResourceGeneCoverage/src/org/wikipathways/Stats.java (PDF) [file pcbi.1004085.s003.pdf]

	**unique genes (total)**	**unique genes with UniProt mapping (protein coding)**
**Ensembl (01-07-2013)**	62,893	22,420
**Reactome (11-09-2014)**	7,771	7,771
**KEGG (11-09-2014)**	7,756	7,500
**WikiPathways (11-09-2014)**	5,673	5,375
**All pathway databases (11-09-2014)**	11,173	10,769
**Percentage**	17.76%	48.03%

Table S2. Gene coverage in pathway databases. All numbers were calculated with the BridgeDb mapping database build on 1 July 2013 (http://bridgedb.org/data/gene_database/ → Hs_Derby_20130701.bridge). We used the Reactome, KEGG and WikiPathways webservices to retrieve the gene lists and map them all to Ensembl identifiers. We only included genes that can also be mapped to UniProt to focus on protein coding genes. This gives a basic indication of the gene coverage in the pathway databases. The scripts for the calculations can be downloaded from https://github.com/mkutmon/wp-scripts/blob/master/PathwayResourceGeneCoverage/src/org/wikipathways/Stats.java
