# Supplementary material for: PathVisio 3: An Extendable Pathway Analysis Toolbox
Source: PLoS Comput Biol. 2015 Feb 23;11(2):e1004085. doi: 10.1371/journal.pcbi.1004085 (PMC4338111; doi:10.1371/journal.pcbi.1004085)
Supplement: S1 Text — (PDF) [file pcbi.1004085.s004.pdf]

Instructions for building and running PathVisio from source

1. Retrieve the latest version of the PathVisio source code from the SVN repository:

   PathVisio 3.1.3
   http://svn.bigcat.unimaas.nl/pathvisio/branches/milestone_36.x/

2. Run ant (http://ant.apache.org/) in the pathvisio main directory.

   This takes a few seconds to build all the individual modules.
   Requirement: Java 1.6 or higher

3. Start PathVisio by either running pathvisio.sh (on Linux and Mac OS) or pathvisio.bat (on Windows)

Instructions for running PathVisio using binary installation

1. Download the binaries for PathVisio 3.1.3:

   http://pathvisio.org/data/releases/3.1.3/pathvisio_bin-3.1.3-r3968.zip

2. Unzip the zip file

3. Start PathVisio by either running pathvisio.sh (on Linux and Mac OS) or pathvisio.bat (on Windows)
