# Supplementary material for: PathVisio 3: An Extendable Pathway Analysis Toolbox
Source: PLoS Comput Biol. 2015 Feb 23;11(2):e1004085. doi: 10.1371/journal.pcbi.1004085 (PMC4338111; doi:10.1371/journal.pcbi.1004085)
Supplement: S2 Text — (PDF) [file pcbi.1004085.s005.pdf]

PathVisio example data and tutorials

On the PathVisio website (http://www.pathvisio.org) we provide documentation and tutorials for new users. The tutorials also contain example datasets that can be used to find out how PathVisio works.

Currently we provide four tutorials (http://www.pathvisio.org/documentation/tutorials/):

1. **Drawing pathways**

This tutorial includes step by step instructions on how to draw a pathway.

2. **Analyzing experimental data with PathVisio**

This tutorial shows how to import experimental data, how to visualize the data on pathway diagrams and how to perform pathway statistics in PathVisio.
An example mouse dataset and mouse pathway collection is used.

3. **Visualizing multi-omics visualization**

This tutorial was created as supplementary data for a publication using PathVisio (van Iersel *et al*, 2014). A transcriptomics and proteomics dataset are integrated and visualized together on the pathway diagrams.

4. **WikiPathways plugin for PathVisio**

PathVisio is the pathway editor using in the community curated pathway database WikiPathways. Besides the applet, we provide a plugin for PathVisio that allows users to search, browse, update and upload new pathways to WikiPathways.
